# Living with diabetes: a group-based self-management support programme for T2DM patients in the early phases of illness and their partners, study protocol of a randomised controlled trial

**DOI:** 10.1186/1472-6963-14-144

**Published:** 2014-04-01

**Authors:** Anne L van Puffelen, Mieke Rijken, Monique JWM Heijmans, Giel Nijpels, Guy EHM Rutten, François G Schellevis

**Affiliations:** 1 NIVEL, Netherlands Institute for Health Services Research, PO Box 1568, 3500 BN Utrecht, The Netherlands; 2 Department of General Practice & Elderly Care Medicine, EMGO Institute for Health and Care Research, VU University Medical Center, Amsterdam, The Netherlands; 3 Julius Center for Health Sciences and Primary Care, UMC Utrecht, Utrecht, The Netherlands

**Keywords:** Diabetes, Randomised controlled trial, Illness perceptions, Partner support, Self-management, Health-related quality of life

## Abstract

**Background:**

The present article presents the protocol for a randomised controlled trial to test the effectiveness of a group-based self-management support programme for recently diagnosed type 2 diabetes mellitus (T2DM) patients (one to three years post-diagnosis) and their partners. The course aims to support T2DM patients and their partners in successfully integrating diabetes care into their daily lives and hereby enhance self-management and diabetes-specific health-related quality of life. The content of the course is based on the Common-Sense Model of Self-Regulation (CSM). Furthermore, principles from the Social Cognitive Theory (SCT) and social support theories are integrated.

**Methods/Design:**

We aim to recruit 160 recently diagnosed T2DM patients and their partners from general practices in six different regions in the Netherlands. Patients need to be diagnosed with T2DM for one to three years and have to experience some degree of diabetes-related difficulties, as measured with a three-item screener. Participating patients and their partners are randomly allocated to the intervention or control condition. Participants in the intervention condition receive three monthly group sessions and a booster session three months later. Participants in the control condition receive a single information meeting. Data will be collected at baseline (T0), directly after the programme (T1) and six months post-programme (T2), including: self-management, diabetes-specific health-related quality of life, illness perceptions, attitudes, social support and empowerment. A three-level multilevel model will be used to compare change-scores between the conditions (intervention/control) on each outcome.

**Discussion:**

Our study will be the first to determine whether a group-based support programme based on the CSM is effective in enhancing self-management and diabetes-specific health-related quality of life in recently diagnosed T2DM patients. The important role of patients’ partners in effective diabetes care is also acknowledged in the study.

**Trial registration:**

Netherlands National Trial Register (NTR) NTR3302.

## Background

The prevalence of type 2 diabetes mellitus (T2DM) is increasing to epidemic proportions. Worldwide, more than 300 million people are diagnosed with T2DM and this number is expected to increase with 50% over the next 20 years [1]. Although T2DM usually starts as a mild condition, its chronic and progressive nature, the necessity for considerable lifelong lifestyle changes and serious long-term complications can place a major burden on individuals and their families [2,3], as well as health care systems [4].

Effective diabetes-management by patients has been proven to reduce the chances of serious adverse events [5] and, consequently, maintain quality of life [6,7] and keep health care costs manageable [8]. However, this does require patients to adopt a complex, multifaceted behavioural regimen, comprising the management of symptoms, treatment and lifestyle changes, as well as dealing with the psychological and psychosocial consequences related to the illness. Moreover, these behaviours need to be embedded within existing lifestyles, goals and priorities. Not surprisingly, a fair proportion of T2DM patients perceives the daily management of diabetes to be challenging or even burdensome and experiences difficulties in adequately engaging in self-care activities [9,10], which might consequently impact on quality of life [7].

Recognition of the comprehensiveness of diabetes management has led to the development of many self-management support programmes [11,12]. However, few have taken the specific challenges that may arise during the early phases of living with T2DM into account. Directly from the onset, T2DM patients are required to make lifestyle changes and adhere to treatment recommendations, mostly in the absence of diabetes-related symptoms or complaints. Hence, patients’ motivation to engage in self-management should therefore primarily result from their beliefs on the likelihood of adverse events occurring, as well as beliefs on personal control and effectiveness of treatment in order to prevent these serious undesirable events [13]. However, according to a review by Thoolen et al., [14], recently diagnosed patients tend to downplay the seriousness of their own condition. In addition, patients seem to be primarily concerned with the day-to-day hassles in diabetes management, rather than the possibility of serious complications in the long term. These attitudes and perceptions are likely to contribute to the finding that relatively few patients appear to be adequately engaged in the recommended (changes in) lifestyle behaviours within the first year after diagnosis [14].

In the past decades, patients’ perceptions on illness and treatment were identified to be important precursors for health behaviour change [15]. According to the Common-Sense Model of Self-Regulation (CSM) [16,17], illness perceptions act as a framework for the coping strategies chosen by patients to deal with the illness and are closely related to behavioural adaption, physical recovery and psychological well-being in various chronic illnesses [18]. Moreover, previous studies have shown that illness perceptions and, consequently, health related behaviours and outcomes can be successfully changed by short interventions based on CSM principles [19-21].

Accumulating evidence shows that not just the patients’ illness perceptions, but also the perceptions of partners are of great importance for understanding how patients respond to a chronic illness [22]. Illness perceptions held by partners guide their coping responses to the patients’ illness, including the way to give support to patients. Previous studies in T2DM have shown that social support can enhance as well as hinder self-management behaviours in patients, particularly dietary and exercise behaviours [22,23]. A small number of studies even suggested that partners’ illness perceptions can influence disease outcomes, with negative or incongruent perceptions being associated with worse physical, psychological and social functioning [24,25]. Hence, even though patients themselves are primarily responsible for managing their illness, it seems important that partners are structurally involved in diabetes care.

**Figure 1:**
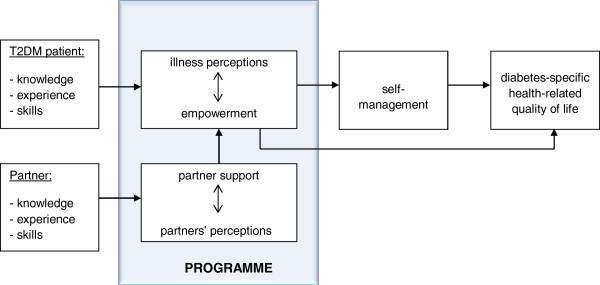
Theoretical model of the programme.

Given the importance to intervene at an early stage in T2DM and the promising results of previous studies based on the CSM, we developed the ‘Living with diabetes’ course: a group-based self-management support programme specifically tailored to T2DM patients and their partners in the first years of living with diabetes. With this new course, we aim to support both patients and partners in successfully integrating diabetes (care) into their daily lives and, hereby, enhancing self-management and diabetes-specific health-related quality of life in T2DM patients. Psychological and social aspects, including perceptions and attitudes, empowerment and social support, are integrated in the course because of their known important role in behaviour change [26] (Figure 1). A more detailed description of the content and underlying theories of the course can be found elsewhere (van Puffelen et al., 2013 submitted).

### Aims and hypotheses

The primary aim of this study is to test the effectiveness of the ‘Living with diabetes’ course on enhancing self-management behaviours and diabetes-specific health-related quality of life in T2DM patients (one to three years post- diagnosis), compared to an attention control condition.

It is hypothesised that participation of T2DM patients in the group-based self-management support programme will result in:

a) Enhanced self-management and diabetes-specific health-related quality of life directly after the programme and at six months post-programme, as compared to an attention control condition.

Furthermore, we hypothesise that participation in the group-based self-management support programme will result in patients:

b) Holding more adaptive illness perceptions and attitudes towards T2DM;

c) Experiencing more activating partner support;

d) Feeling more empowered to manage their condition

Directly after the programme and at six months post-programme, as compared to an attention control condition.

## Methods/Design

### Study design

**Figure 2:**
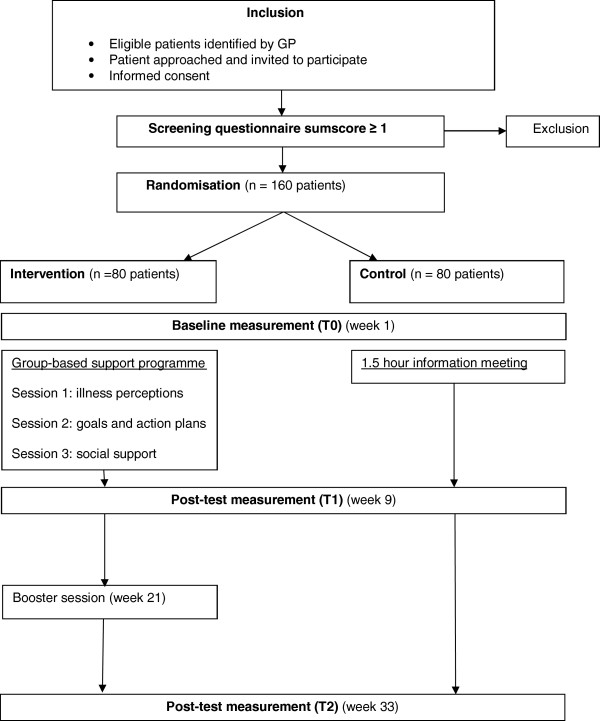
Flow of participants.

The effectiveness of the programme will be evaluated by a randomised controlled trial with two follow-up measurements: immediately after the programme (T1) and six months after T1 (T2) (Figure 2).

### Study population

Patients will be recruited via general practitioners (GPs) who are willing to invite eligible patients to participate in the study. In the Netherlands, all inhabitants are registered in a general practice. Therefore, a representative sample can be drawn.

#### Inclusion criteria

Being diagnosed with T2DM for one to three years, as recorded by their GP.

#### Exclusion criteria

● Not being able to speak, read and/or understand the Dutch language sufficiently according to their GP;

● Having insufficient mental or intellectual capabilities to participate in the study, according to their GP;

● Currently receiving treatment for severe psychological or psychiatric conditions, according to their GP;

● Recently diagnosed with a severe or life-threatening comorbid condition (e.g. cancer, CVA);

● Not experiencing any degree of diabetes-related difficulty or uncertainty, as assessed with a three-item screening questionnaire.

### Recruitment

The study population will be recruited from participating general practices in different regions in the Netherlands (North, West, Southwest and center). Eligible patients are selected from the medical records of the participating general practices and receive a written invitation for participation via their GP. Patients fill in an informed consent form as well as a short screening questionnaire, developed to identify patients who experience at least some degree of difficulty or challenge regarding their T2DM self-management. The questionnaire includes two questions of the Appraisal of Diabetes Scale (ADS) [27] and one statement of the Revised Illness Perception Questionnaire (IPQ-R) [28], assessing diabetes-related uncertainty, coping with diabetes and perceived consequences of diabetes on life. Patients with a total sum score of zero, indicating that they do not experience any difficulties or challenges regarding their diabetes management, are excluded from the study (see Table 1).

**Table 1:** Screening questionnaire

1. How much uncertainty do you currently experience in your life as a result of being diabetic?				
None at all	Slight amount	Moderate amount	Large amount	Extremely large
(0)	(1)	(1)	(1)	amount (1)
2. How effective are you in coping with your diabetes?				
Not at all	Slightly effective	Moderately	Very effective	Extremely effective
(1)	(1)	effective (1)	(0)	(0)
3. My diabetes has major consequences on my life.				
Strongly disagree	Disagree	Neither agree nor	Agree	Strongly agree
(0)	(1)	disagree (1)	(1)	(1)

### Allocation to conditions

After obtaining the signed informed consent form, patients will be randomly allocated to the intervention or control condition. Randomisation will be conducted electronically by a researcher who is not involved in the study.

#### Intervention

Participants allocated to the intervention group are invited to take part in the group-based self-management support programme, together with their partner. Patients who do not have a partner, are instructed to bring a close friend or relative instead. Each course group consists of six to ten patients, accompanied by their partner (or close friend/relative). The course consists of three two-hour monthly meetings and one follow-up meeting (‘booster session’) after three months. The group sessions are led by two trained diabetes nurses or practice nurses and are delivered in medical and community centers in the different regions.

##### Framework

The ‘Living with diabetes’ course is based on the Common-sense Model of Self-Regulation [16,17], the Social Cognitive Theory [29,30] and principles of social support theories [31-33]. Content of the course is derived from previous psychosocial interventions focusing on illness perceptions [19,20,34]. The emphasis of the course is on stimulating beneficial illness perceptions and challenging misconceptions of T2DM in patients and partners. Another important aspect of the course is the enhancement of activating partner support for patients, by exploring patients’ needs for support and discussing supportive interactions with patients and partners. Goal setting and action plan development are used as techniques to improve patients’ empowerment and elicit self-management behaviour change. All sessions are group-based, providing the opportunity for peer modeling, social reinforcement, motivation and emotional support.

##### Materials

Participants receive a handbook with (homework) assignments, and practical and theoretical information about the topics discussed during the sessions. In addition, basic information about diabetes and its treatment is provided in the handbook. For the diabetes or practice nurses who guide the course sessions, a detailed manual has been developed.

##### Pilot

The course was pilot-tested on feasibility and acceptability in November and December 2011. Sixteen T2DM patients and eight partners from a general practice in the region of Utrecht participated in the pilot-study (attrition rate 21.6%). During the pilot, all course sessions were led by a health psychologist in order to evaluate whether the correct psychological models and techniques were used. During the first session, a practice nurse was also present to provide medical information on T2DM. Feasibility and acceptability of the course were explored by means of an evaluation form at the end of the course and by feedback of the participants during the course sessions. Based on the evaluation of the pilot, the manual was adapted and a screening questionnaire was developed for the RCT to ensure that only T2DM patients who experience some degree of difficulties or uncertainties will participate.

##### Training

Prior to the course, the participating nurses receive a four-hour training, led by a health psychologist who was also involved in the development of the course (MH). During this training, the nurses receive information on the underlying theories on which the course is based. Furthermore, the nurses are instructed on how to use the workbook and manual of the course. Assignments of the course are explained in detail and tips and tricks on how to execute these assignments provided. Lastly, first experiences of the pilot and resulting important topics of interest are discussed.

#### Attention control condition

Participants in the attention control condition are invited to a single 1.5 – hour information meeting, together with their partner (or close friend/relative). During this meeting, patients and their partners receive medical information about diabetes (e.g. causes, complications, treatment) from a professor in general practice and diabetes care. The information meeting serves as an attention control condition to control for the attention paid to being diagnosed with T2DM when participating in this study. Hence, the information that patients and partners receive during the information meeting is provided according to the classical didactic method; i.e. providing information that is important from a medical point of view, but not tailored to the specific and more comprehensive needs of the patients.

### Measures

Patients fill in a questionnaire at baseline (T0), immediately after the programme (T1) and six months post-programme (T2) to assess the effectiveness of the programme on the longer term. The primary outcome measures are self-management and diabetes-specific health-related quality of life. Secondary outcomes are illness perceptions, attitudes towards diabetes, partner support and empowerment.

#### Primary outcome measures

Self-management is measured by using the revised Summary of Diabetes Self-Care Activities measure (SDSCA) [35]. The revised SDSCA contains 11 items, measuring six separate domains: general diet (2 items), specific diet (2 items), exercise behaviours (2 items), glucose monitoring (2 items), foot care (2 items) and smoking (1 item). Ten items are rated on an eight -point Likert scale, measuring the number of days a certain self-care behaviour is performed during the last week (0–7 days). The 11^th^ item measures smoking (yes/no) and the number of cigarettes smoked. Each of the domains is measured separately. The revised SDSCA shows an adequate internal consistency and test-retest reliability and is sensitive to change. The measure has been validated against other measures of diet and exercise [35].

Diabetes-specific health-related quality of life is assessed by the Problem Areas in Diabetes scale (PAID) [36], measuring diabetes-related emotional distress. The PAID consists of 20 items on a five -point Likert scale, ranging from 0 (not a problem) to 4 (a serious problem). Scores are transformed into a 0–100 scale for interpretation, with higher scores indicating greater diabetes-related emotional distress. The PAID has a strong concurrent and discriminant validity [37], has been proven to be responsive to change [38] and has been validated for Dutch T2DM patients [36,37].

#### Secondary outcome measures

Cognitive and emotional illness perceptions are assessed with the IPQ-R [28]. The first section of the IPQ-R measures different symptoms experienced by patients and whether they believe these symptoms are caused by their diabetes (identity scale; 14 items). The second section of the IPQ-R consists of seven subscales, measuring ‘time-line acute/chronic’ (6 items); ‘time-line cyclical’ (4 items); ‘consequences’ (6 items); ‘personal control’ (6 items); ‘treatment control’ (5 items); ‘coherence’ (understanding of T2DM, 5 items) and ‘emotional representation’ (6 items). In the third section, patients’ causal believes (18 items) are measured. The ‘identity scale’ is measured dichotomously (yes/no). All other items are measured on a five-point Likert scale ranging from 1 (strongly disagree) to 5 (strongly agree). The subscales of the IPQ-R have a good internal consistency and an acceptable test-retest stability [28].

Attitudes towards diabetes are measured with the Diabetes Attitude Scale (DAS-3) [39]. The DAS-3 consists of five subscales, measuring perceived seriousness (7 items); psychosocial impact (6 items); patient autonomy (8 items); value of tight control (7 items) and need for special training (5 items). The items are measured on a five-point Likert scale ranging from 1 (strongly disagree) to 5 (strongly agree). The DAS-3 is considered a reliable and valid general measure of diabetes-related attitudes across different groups of patients and health care professionals [39].

Patients’ perceptions of partner support are assessed by using a questionnaire developed by Buunk, Sanderman, and Nieuwland [40], which measures three different dimensions of partner support; active engagement (5 items); protective buffering (8 items) and overprotection (6 items). The items are measured on a five-point Likert scale ranging from 1 (never) to 5 (always). The three subscales have a moderate to good internal consistency [40].

Patient empowerment is assessed by the Dutch Diabetes Empowerment Scale (Dutch DES-20) [41]. The questionnaire consists of five subscales, assessing dissatisfaction and goal achievement (6 items), coping and motivation (4 items), obtaining support (3 items), overcoming barriers (4 items) and determining suitable methods (3 items) in a five-point Likert scale, ranging from 1 (totally disagree) to 5 (totally agree). The Dutch DES-20 was found to be a reliable and valid instrument [41].

### Sample size

The sample size calculation is based on detecting a clinically relevant change on the PAID, as a result of the programme. Since there is no consensus on the minimal important difference (MID) on the PAID, we decided to set the MID at half a standard deviation (SD); a commonly used solution when scores have no direct interpretation and no clinical results exist to determine a relevant percentage [42]. In Dutch T2DM patients, the SD found on the PAID was 20, with a mean score of 22.5 points (scale 0–100) [36]. To establish a 10-point difference with the power set at 80% and the α at .05 (two-sided), 63 patients are needed in each condition (intervention/control). However, taking the clustering of patients within groups into account, an oversampling of 15% is needed to conduct multi-level analyses. When accounting for an additional drop-out of 10%, 2 × 80 patients will have to be recruited.

### Statistical analysis

The study is a two-arm randomised controlled trial with repeated measures over time and continuous outcome variables. Descriptive statistics (mean values and frequencies) will be calculated to evaluate the scores on primary and secondary outcome measures on T0, T1 and T2 separately. The effectiveness of the programme will be analysed by a three-level multilevel model: groups, patients and measurements (T0, T1, and T2). This type of analysis allows us to both test the main effectiveness of the condition, its effectiveness over time, as well as the interaction effects of condition (intervention/control) × time. By including groups as a separate level in the analysis, possible effects of the different regions, course leaders and group climate are corrected for. Data will be analysed according to the intention-to-treat principle. All analyses will be performed using MLwiN.

### Ethical approval

The protocol, information letters and informed consent form of the study were approved by the Medical Ethical Committee of the VU University Medical Center Amsterdam.

## Discussion

This article describes the design of the ‘Living with diabetes’ study: a study testing the effectiveness of a group-based self-management support programme for people known to be diagnosed with T2DM for one to three years and their partners. The content of the course is based on principles of the CSM, SCT and social supportive theories and is specifically designed to build more adaptive (activating) illness perceptions and attitudes, increase empowerment, stimulate activating social support and, consequently, enhance self-management and diabetes specific health-related quality of life in recently diagnosed T2DM patients.

Previous research already emphasised the importance of the integration of patients’ and partners’ illness perceptions in self-management interventions, because of their ability to change and their close link to health behaviours and outcomes [15,19-21]. With this study, we will contribute to the literature by providing insight into the effectiveness of a group-based method to build and alter illness perceptions in patients with chronic illness, rather than an individual programme. Furthermore, to our knowledge, we are the first to conduct such programme in T2DM patients and partners in the first years of living with T2DM.

A particular strength of the study is that the programme ‘Living with diabetes’ is well grounded in theory. Major psychological models on behaviour change, such as Leventhal’s Common Sense Model [16,17] and Bandura’s Social Cognitive Theory [29,30] provide the framework for the course. By incorporating social support theories and actively involving the patients’ partners, we account for the influence of social support on self-management behaviours in patients. Group discussions provide patients with the opportunity to share experiences and learn from others through peer modelling and peer support [43]. Consequently, the course goes beyond the mere provision of information and skills training and starts explicitly from both patients’ and partners’ experiences, needs and concerns. In addition, this study specifically focusses on patient important outcomes rather than solely medical outcomes (e.g. HbA1c), comprising emotional, cognitive and behavioural outcomes.

The current study also poses a number of challenges and drawbacks. We foresee a few potential threats to reliability and generalisability of the study. First, a selection of participating patients in the study is expected, as a result of a selective non-response of patients of older age and patients from the non-western origin. Consequently, specific target groups might be missed and generalisability of the results of this study limited. Furthermore, a possible selection bias might also be found among the GPs in the study. Participating GPs will probably represent a group more open to research and innovation and may also be more motivated to improve diabetes care. Consequently, their patients might already receive various educational or support programmes which may negatively impact on participation willingness. In order to keep non-response and drop-out rates as low as possible, personalised invitation letters from GPs and reminders to initial non-responders will be sent. Furthermore, the course sessions and information meetings will be organised in easily accessible locations in the area of the participating patients and GPs. Finally, a practical challenge is also foreseen in the group-based format of the course. In spite of the fact that group-based sessions pose many advantages, they are more difficult to organise and cannot be completely adapted to individuals needs and preferences (e.g. time, location, topics discussed), which may result in increased (selective) drop-out. Furthermore, we emphasise the importance of creating and keeping a positive and stimulating group climate during the course sessions. Dominant and/or negative group members can negatively influence the group climate and interactions, and consequently, the effectiveness of the programme. Therefore, we will recruit diabetes nurses and practice nurses who already have experience in leading group-based courses and extra attention will be paid on how to deal with dominant group members during the training.

The results of this RCT will provide valuable information on the effectiveness and feasibility of group-based self-management support programmes, focusing on illness perceptions and social support. The course is well suited for implementation in a primary health care setting. The course is fully manualised and supported by a training to ensure the possibility of replication. Furthermore, the group-based setting of the course is less time and money consuming than individual support programmes. Hence, if proven effective, the course can be utilised by general practices and diabetes care groups as an addition to the individual patient education provided by health care professionals and already available patient education programmes in T2DM. First results of the study are expected in the spring of 2014.

## Competing interests

The authors declare that they have no competing interests.

## Authors’ contributions

AP wrote the manuscript. The study design and research proposal were developed by FS, MR, GR and GN. The course was developed by MH and AP. All authors read and approved the final manuscript.

## Pre-publication history

The pre-publication history for this paper can be accessed here:

http://www.biomedcentral.com/1472-6963/14/144/prepub
